# Biochemical characterization of transmembrane proteins (TMEMs) in the ciliary transition zone

**DOI:** 10.1186/2046-2530-4-S1-P75

**Published:** 2015-07-13

**Authors:** E Shoaib, Z Abdelhamed, K Szymanska, S Bell, E Morrison, C Johnson

**Affiliations:** 1 Leeds Institute of Biomedical & Clinical Sciences, University of Leeds, Leeds, UK

## Objective

Ciliopathies are a group of heterogeneous disorders caused by mutations in proteins associated with primary cilia. Many proteins that are mutated in ciliopathies Joubert syndrome (JBTS), Meckel-Gruber syndrome (MKS) and nephronophthisis (NPHP) are localized to the transition zone (TZ), a compartment of the proximal region of the cilium. In particular, a protein complex known as the "MKS-JBTS module" contains many transmembrane proteins (TMEMs) that are mutated in these conditions. Here, we aim to understand the role of the ciliary proteins TMEM67, TMEM138, TMEM216, TMEM237, TMEM17 and TMEM231 by characterizing their biochemical functions and potential interactions. We hypothesize that pathogenic missense mutations disrupt the putative TMEM complex or localization to the TZ. Furthermore, we aim to identify new interacting proteins of TMEMs, including potential ligands of the orphan receptor TMEM67.

## Methods

The "Gateway" cloning system tagged TMEM138, TMEM216, TMEM237, TMEM17, and TMEM231 at the *N-*termini with GFP, TAP (streptavidin/FLAG) or the FLAG epitope. Constructs were exogenously expressed in ciliated mIMCD3 cells and expression confirmed by western blotting or immunofluorescence confocal microscopy. A construct for the *N*-terminal extracellular domain of TMEM67 (pSecTagA2-TMEM67-Nt) was made for secreted protein expression.

## Results

IF microscopy confirmed the sub-cellular localization of tagged TMEM proteins to the cell membrane and basal body, and co-immunoprecipitations show that TMEM237 interacts with TMEM138 or TMEM216. High levels of TMEM67-Nt protein have been purified for use in future biochemical experiments.

## Conclusions

Biochemical methods for the functional characterization of ciliary TMEM proteins should provide insights into the molecular pathogenesis of ciliopathy mutations.

